# Bilateral varicocele leads to ferroptosis, pyroptosis and necroptosis of human spermatozoa and affects semen quality in infertile men

**DOI:** 10.3389/fcell.2023.1091438

**Published:** 2023-02-02

**Authors:** Tie Cheng Sun, Dong Mei Li, Hong Yu, Ling Li Song, Yan Jun Jia, Li Lin, Shan Jie Zhou

**Affiliations:** ^1^ Reproductive Medical Center, Department of Obstetrics and Gynecology, Peking University International Hospital, Beijing, China; ^2^ HLA Laboratory, Beijing Red Cross Blood Center, Beijing, China

**Keywords:** bilateral varicocele, ferroptosis, pyroptosis, necroptosis, semen quality

## Abstract

**Purpose:** This study explored the effects of bilateral varicocele on male semen quality in infertile men and the molecular mechanisms involving ferroptosis, pyroptosis and necroptosis signaling pathways.

**Methods:** Totally, 20 healthy males and 26 patients with bilateral varicocele receiving infertility treatment were enrolled. Semen samples were collected. Basic semen parameters, acrosome integrity and membrane integrity, mitochondrial membrane potential (MMP) and apoptosis rate were compared. Levels of reactive oxygen species (ROS), iron, glutathione (GSH), total superoxide dismutase (T-SOD), and, Catalase (CAT), were detected in human seminal plasma. Relative mRNA expression of Ca ^2+^-independent phospholipases A2 beta (*iPLA 2β*), P53, Zinc finger E-box binding homeobox 1 (*ZEB1*) and GSH-dependent peroxidase 4 (*GPX4*) were evaluated. Relative protein expression was determined for GPX4, receptor interacting serine/threonine kinase 1 (RIPK1) and receptor interacting serine/threonine kinase 3 (RIPK3), as well as pyroptosis markers of Gasdermin E (GSDME) and heat shock protein 90 (HSP 90).

**Results:** The results revealed that the bilateral varicocele group had significantly higher abnormalities (sperm progressive rate and sperm motility) compared to the control group. Meanwhile, compared to control group, GSH, T-SOD, and CAT levels were reduced in the bilateral varicocele group (*p* < 0.05). However, the level of ROS and iron were significantly increased (*p* < 0.05). Relative mRNA expression of *P53*, *iPLA 2β*, *ZEB1*, and *GPX4* were reduced. In addition, ROS exposure activated ferroptosis-related signal pathways. RIPK1, RIPK3, GSDME and HSP 90 were increased in bilateral varicocele group. ROS exposure affected signaling pathways related to ferroptosis, necrosis and pyroptosis in human spermatozoa.

**Conclusion:** Bilateral varicocele leads to ferroptosis, pyroptosis and necroptosis of human spermatozoa and affects semen quality in infertile men.

## 1 Introduction

Infertility has become a global health problem in recent years and affects 10%–15% of couples of reproductive age (Mazzilli et al., 2021). Of all infertility cases, male factor infertility accounts for approximately 20%–70%, and declined sperm count and semen quality have been widely reported in recent years (Cohen et al., 2021; Mazzilli et al., 2021). The common reason for male infertility is impaired spermatogenesis or sperm capability, which might be affected by genetic disorders, genital tract infections, medical interventions, environmental contamination, and lifestyle behaviors (Ou et al., 2020; Boedt et al., 2021). Bilateral/unilateral varicocele is also considered a risk factor for male infertility, because it can directly or indirectly damage spermatogenesis and/or sperm function (Panner Selvam et al., 2020). Although many studies have indicated the roles of sperm DNA damage and apoptosis in patients with bilateral varicocele (Panner Selvam et al., 2020; Jeremias et al., 2021), the cellular mechanism underlying varicocele-related male infertility remains unknown. In bilateral varicocele, there are increased levels of cellular reactive oxygen species (ROS), nitric oxide (NO) and free radicals, which can potentially lead to oxidative stress (Dobanovacki, 2010; Panner Selvam et al., 2019). Therefore, this might be the main reason for the decline of semen quality in bilateral varicocele, which has also been reported in previous studies (Agarwal et al., 2015; Panner Selvam et al., 2019). In addition, it has also been reported that ROS, NO and free radicals can affect basic semen parameters (Agarwal et al., 2014; Panner Selvam et al., 2019; Sun et al., 2020). Based on these findings, we conclude that bilateral varicocele could produce ROS, NO and free radicals, which promote apoptosis and programmed death, but little is known regarding the potential molecular mechanisms.

Ferroptosis is an iron-dependent, non-apoptotic cell death, which was firstly observed by Dixon et al., in 2012 (Dixon et al., 2012). Ferroptosis is dependent on accumulated iron, lipid peroxidation, and excessive accumulation of lethal ROS (Yang and Stockwell, 2016). The classic regulator of ferroptosis is glutathione-dependent peroxidase 4 (GPX4), which is the major protector of cellular peroxidation damage (Hayano et al., 2016). One of the main mechanisms of GPX4 inactivation is the deprivation of glutathione (GSH) (Wehn et al., 2021). GSH maintains redox homeostasis by acting as a reductive substrate for ROS-detoxifying enzymes (Wehn et al., 2021). Ferroptosis is different from apoptosis, autophagy, and necrosis in morphology, metabolism, and biochemistry. It has been shown that the ferroptosis is caused by the imbalance between ROS production and peroxidation-antioxidant system (Han et al., 2021). Importantly, Imai et al. showed that GPX4 was strongly expressed in the testis and spermatozoa, and that 30% of the infertile men diagnosed with oligoasthenozoospermia had significantly lower GPX4 expression in spermatozoa (Imai et al., 2001). Furthermore, the depletion of GPX4 in spermatocytes could cause severe abnormalities in spermatozoa (sperm concentration and motility), suggesting that GPX4 plays a crucial role in spermatogenesis (Imai et al., 2009). Bromfield et al. also demonstrated a strong and consistent relationship between ferroptosis and spermatids (Bromfield et al., 2019). In addition to the relationship of ferroptosis with phospholipid oxidation, pyroptosis and necroptosis, the relationship between phospholipid oxidation and fertility have also been studied (Madi et al., 2021; Hasani et al., 2022; Liu et al., 2022; Zhou et al., 2022). Previous studies have found that markers of Ca ^2+^-independent phospholipases A2 beta (iPLA2β), receptor interacting serine/threonine kinase 1 (RIPK1) and receptor interacting serine/threonine kinase 3 (RIPK3), gasdermin E (GSDME), and heat shock protein 90 (HSP 90) are associated with ferroptosis, pyroptosis and necroptosis (Chen et al., 2021; Han et al., 2021; Wehn et al., 2021). However, these findings have not been detected in human spermatozoa, especially patients with bilateral varicocele. We suppose that these may also be detected in bilateral varicocele, which might reduce the semen quality *via* inducing lipid peroxidation and the further occurrence of ferroptosis, pyroptosis and necroptosis.

Therefore, in this study, we aim to investigate the potential mechanism of the sperm impairment caused by bilateral varicocele. We first collected the semen samples from bilateral varicocele patients and evaluated the semen quality *via* computer-aided sperm analysis. In addition, the markers of ferroptosis, pyroptosis and necroptosis were also detected in human spermatozoa, including GPX4, RIPK1 and RIPK3, GSDME and HSP 90.

## 2 Materials and methods

### 2.1 Reagents

Sperm DNA fragmentation assay kit was purchased from Zhejiang Xingbo Biotechnology Co., Ltd. (Ningbo, China); Acrosome integrity assay kit, membrane integrity assay kit, ROS assay kit, mitochondrial membrane potential (MMP) assay kit and apoptosis assay kit were purchased from Celula, Medical Technology Co., Ltd. (Chengdu, China); GSH, total superoxide dismutase (T-SOD), Catalase (CAT) activities kit and iron assay kit were purchased from Nanjing Jiancheng Institute of Bioengineering (Jiangsu, China); RNA and protein assay kit were obtained from TIANGEN Biochemical Technology Co. Ltd. (Beijing, China); BCA protein Assay kit (Solarbio, China); Rabbit antibodies against GPX4, RIPK1, RIPK3, and, pyroptosis markers of GSDME and HSP 90 were all obtained from Cell Signaling Technology Biological Reagent Co., Ltd. (Shanghai, China).

### 2.2 Study participants

The study was approved by the Ethics Committee of the Peking University International Hospital. This study involving human subjects was performed in accordance with guidelines of the Declaration of Helsinki. Informed patient consent was obtained from the participants who visited the reproductive medicine center in the Peking University International Hospital for infertility treatment during January 2021 to June 2021.

### 2.3 Semen collection and assessment

Totally, 26 males with bilateral varicocele (grade 2 or grade 3) and 20 healthy males were enrolled. All participants were examined in the standing positions, and the diagnosis standards and methods of varicocele included was visible swelling of the scrotum, and palpation of the spermatic cord at rest and during the Valsalva maneuver. Moreover, the ultrasound criteria for diagnosing a varicocele was spermatic vein diameter ≥2 mm and retrograde blood flow. There three grades of varicocele by the clinical grading system: grade 0 (subclinical): non-palpable and visualized only by Color Doppler ultrasound (CDUS); grade 1: palpable only with Valsalva maneuver; grade 2: easily palpable but not visible; and grade 3: easily visible. According to abovementioned criteria, varicocele on either side of the spermatic vein was excluded in 20 control males (Wein, 2007).

Semen samples were collected in sterile containers from healthy males and bilateral varicocele patients by masturbation after 2–7 days of sexual abstinence. After liquefaction, the characteristics of sperm concentration, pH, sperm motility, and sperm morphology were examined using a computer-assisted semen analyzer (WL-9000 sperm analyzer, Beijing Weili New Century Technology Development Co., LTD., Beijing, China) according to WHO guidelines (Sun et al., 2018). All semen samples were centrifuged at 500 g for 5 min. Then, the seminal plasma and spermatozoa were separated and stored at −80°C until analysis.

### 2.4 Intracellular ROS

Intracellular ROS was detected using propidium iodide (PI) and dichloro-dihydro-fluorescein diacetate (DCFH-DA) fluorescence staining (Sperm ROS Detect Kit™, Celula, China) (Yu et al., 2019). Briefly, the spermatozoa were added with DCFH-DA at a final concentration of 40 *μ*M and PI at a final concentration of 10 *μ*M. After incubation for 25 min at 37°C, the samples were centrifuged at 800 g for 3 min. All analyses were performed by flow cytometry (50 μL, 5000 particles and max 5 min, Sparrow, Celula, Chengdu, China).

### 2.5 Sperm chromatin structure assay

The sperm chromatin structure assay required a minimum of 10 μL semen samples with a concentration of ≥0.5 million/mL. Firstly, the frozen specimens were thawed in a 37°C water bath and re-suspended in TNE (Tris, NaCl and EDTA) buffer at the ratio of 1:9 (μL). Subsequently, the samples were treated with 400 μL of acid (pH1.20) for 30 s to denature DNA at the sites of strand breaks. Thirdly, acridine orange staining solution was added to stain the single-strand DNA breaks (presenting with red fluorescence) and double-strand DNA breaks (presenting with green fluorescence). At least 5,000 sperm cells were analysed per sample within 5 min using flow cytometry. The sperm fragmentation index was calculated as red fluorescence/(red and green fluorescence).

### 2.6 Sperm acrosome integrity

Sperm acrosome integrity was determined as described in a previous study (Yániz et al., 2017). Briefly, spermatozoa were stained with the fluorochromes of acrosome integrity assay kit (The labelling mixture included PI, Hoechst 33342, and carboxyfluorescein diacetate). At least 5000 spermatozoa were examined using flow cytometry.

### 2.7 Sperm membrane integrity

The integrity of human spermatozoa sperm membrane was stained with PI (Sperm Membrane Integrity Kit™, Celula, China). This fluorescent probe has a high affinity for sperm DNA but is non-permeable to the sperm plasma membrane, only staining damaged (non-viable) sperm plasma membranes. Sperm with membrane damage passes through the FL2 channel and emits red fluorescence. Briefly, 2.5 μl PI (1 mg/ml) was added to 10 μl sperm samples, and then 500 μl 1% (w/v) NaCl was added. After incubation in the dark for 5 min, the samples were detected by flow cytometry (50 μL, 5000 particles and max 5 min). Sperm plasma membrane integrity was defined as the percentage of PI-negative cells (membranes intact) per sample per treatment.

### 2.8 MMP

MMP was evaluated using the specific probe JC-1 (Sperm MMP Staining Kit™, Celula, China) according to the manufacturer’s instruction. The semen samples were centrifuged at 500× g for 20 min and the supernatant was removed. JC-1 is a lipid cationic fluorochrome that is widely used as a fluorescent probe for the detection of MMP. When the MMP value is high, JC-1 accumulates in the mitochondrial matrix and emits orange fluorescence (585 nm). Conversely, when the MMP value is low, JC-1 accumulates less in the mitochondrial matrix, and emits green fluorescence (525 nm) as a monomer. The MMP was calculated using fluorescence ratio of JC-1-aggregates (red) to JC-1-monomer (green).

### 2.9 Apoptosis

The sperm apoptosis was evaluated using the fluorescent probes Annexin V-FITC (Sperm Apoptosis Staining Kit™, Celula, China) according to the manufacturer’s instruction. Briefly, 45 µL of human spermatozoa (>5 * 10^6^/mL) were incubated with Annexin-V, H-42, and Annexin V-FITC for 60 min at room temperature in the dark. A total of 5000 sperm cells were analyzed by flow cytometry (50 μL, 5000 particles and max 5 min). Apoptosis was calculated using the following formula: Annexin V-FITC positive/H-42 positive and PI negative.

### 2.10 Iron assay

The relative iron concentration in cell lysates was assessed using the Iron Assay Kit (Nanjing Jiancheng Institute of Bioengineering (Jiangsu, China) according to the manufacturer’s instructions (Sun et al., 2015).

### 2.11 Measurement of antioxidant enzyme activities

Spermatozoa were prepared as 10% cell lysates in normal saline and centrifuged at 3000 rpm at 4°C for 15 min. The supernatant was collected. The relative concentrations of GSH, T-SOD, and CAT were assessed using the corresponding kits (Nanjing Jiancheng Bioengineering Institute) according to the manufacturer’s instructions (Deng et al., 2017).

### 2.12 RNA extraction and quantitative real-time PCR

Total RNA was extracted from human spermatozoa using a universal RNA extraction kit (TIANGEN Biochemical Technology Co. Ltd., Beijing, China) according to the manufacture’s protocol. Subsequently, cDNA was obtained by reverse transcription using a commercial kit (TIANGEN Biochemical Technology Co. LTD., Beijing, China). Abundance of mRNA transcripts encoding *iPLA 2β*, *P53*, Zinc finger E-box binding homeobox 1 (*ZEB1*), *GPX4*, and *GAPDH* were measured by quantitative real-time PCR. Primer sequences are shown in Table 1.

**TABLE 1:** Sequences of quantitative real-time PCR primers.

Gene	Forward primer	Reverse primer
*GPX4*	GAG​GCA​AGA​CCG​AAG​TAA​ACT​AC	CCG​AAC​TGG​TTA​CAC​GGG​AA
*ZEB1*	CCT​GTC​CAT​ATT​GTG​ATA​GAG​GC	ACC​CAG​ACT​GCG​TCA​CAT​GT
*iPLA2β*	GCA​ATG​CTC​GGT​GCA​ACA​T	ACA​CCC​CTT​CTG​AGA​GAA​CTT​CA
*P53*	ATGAAGCTCCCAGAATGC	GGGCCGCCGGTGTAG
*GAPDH*	GAA​GGT​GAA​GGT​CGG​AGT​C	GAA​GAT​GGT​GAT​GGG​ATT​TC

### 2.13 Western blot analysis

Total protein from human spermatozoa were extracted with RIPA lysis buffer on ice and Protein Extraction Assay kit (TIANGEN Biochemical Technology Co. Ltd., Beijing, China). BCA protein Assay kit (Solarbio, China) was used to quantitate concentration of protein in the supernatant. Equivalent amounts of protein were separated on 10%–15% SDS-polyacrylamide gels and blotted onto a nitrocellulose membrane. After blocking with 5% skimmed milk for 2 h at room temperature in TBS with 0.1% Tween-20, membranes were probed with GPX4 (1:5000 dilution), GSDME (1:5000 dilution), RIPK1 (1:5000 dilution), RIPK3 (1:5000 dilution), GAPDH (1:5000 dilution), and HRP conjugated IgG antibodies (1:10,000 dilution). Relative band intensity was then determined by ImageJ 1.53 software (National Institutes of Health, America).

### 2.14 Statistical analysis

All experiments were performed independently for at least three times. The independent sample *t*-test was used to analyze numerical data between two groups. Student t-test was used to analyze significant differences between two groups. Data analysis was performed by using Statistical Program for Social Sciences (SPSS) software, version 22.0 (IBM Corporation, Armonk, NY, United States), and a *p*-value <0.05 indicated statistical significance.

## 3 Results

### 3.1 Semen parameters of study participants

This study included 20 healthy males aged 29–34 years old (control group) and 26 patients with bilateral varicocele aged 28–40 years old (patient group) (Table 2). The duration of abstinence (3.77 d vs. 3.7 d), PH (7.35 vs. 7.3) and sperm counts (231.94 *10^6^ ells vs. 177.67 *10^6^ cells) between the two groups were not significantly different (*p* > 0.05). However, there were significant differences in other basic semen parameters, including semen volume (2.58 mL vs. 3.87 mL), sperm concentration (87.25 *10^6^/mL vs. 53.32 *10^6^/mL) and normal morphology (1.4% vs. 0.85%) (*p* < 0.05). Additionally, sperm progressive rate (47.68% vs. 20.83%), sperm motility (58.46% vs. 24.38%) and sperm DNA fragmentation index (10.78% vs. 22.07%) between the two groups were significantly different (*p* < 0.001) according to the 2010 WHO criteria (Murray et al., 2012).

**TABLE 2:** The semen parameters of 20 control and 26 patients.

Parameter	Control	Patients	*p*
N	20	26	
Age (y)	32.96 ± 3.67	34.8 ± 6.42	0.227
Duration of abstinence (day)	3.77 ± 1.24	3.7 ± 1.49	0.864
Semen volume (mL)	2.58 ± 0.82	3.87 ± 2.5	0.017
PH	7.35 ± 0.32	7.3 ± 0.25	0.593
Sperm concentration (* 10^6^)	87.25 ± 56.61	53.32 ± 31.67	0.021
Sperm count (* 10^6^ cells/mL)	231.94 ± 191.98	177.67 ± 119.93	0.247
Sperm progressive rate (PR, %)	47.68 ± 13.23	20.83 ± 11.26	**0.001**
Sperm motility (PRNP, %)	58.46 ± 15.43	24.38 ± 13.53	**0.001**
Normal morphology (%)	1.4 ± 0.96	0.85 ± 0.65	0.032
Sperm DNA fragmentation index (DFI, %)	10.78 ± 4.7	22.07 ± 12.94	**0.001**
High DNA stainability (HDS, %)	7.95 ± 5.0	12.6 ± 9.14	0.063

Note: Data are presented as mean ± SD; Bold values indicates a *P* of not less than 0.5.

### 3.2 Acrosome integrity and membrane integrity

**FIGURE 1:**
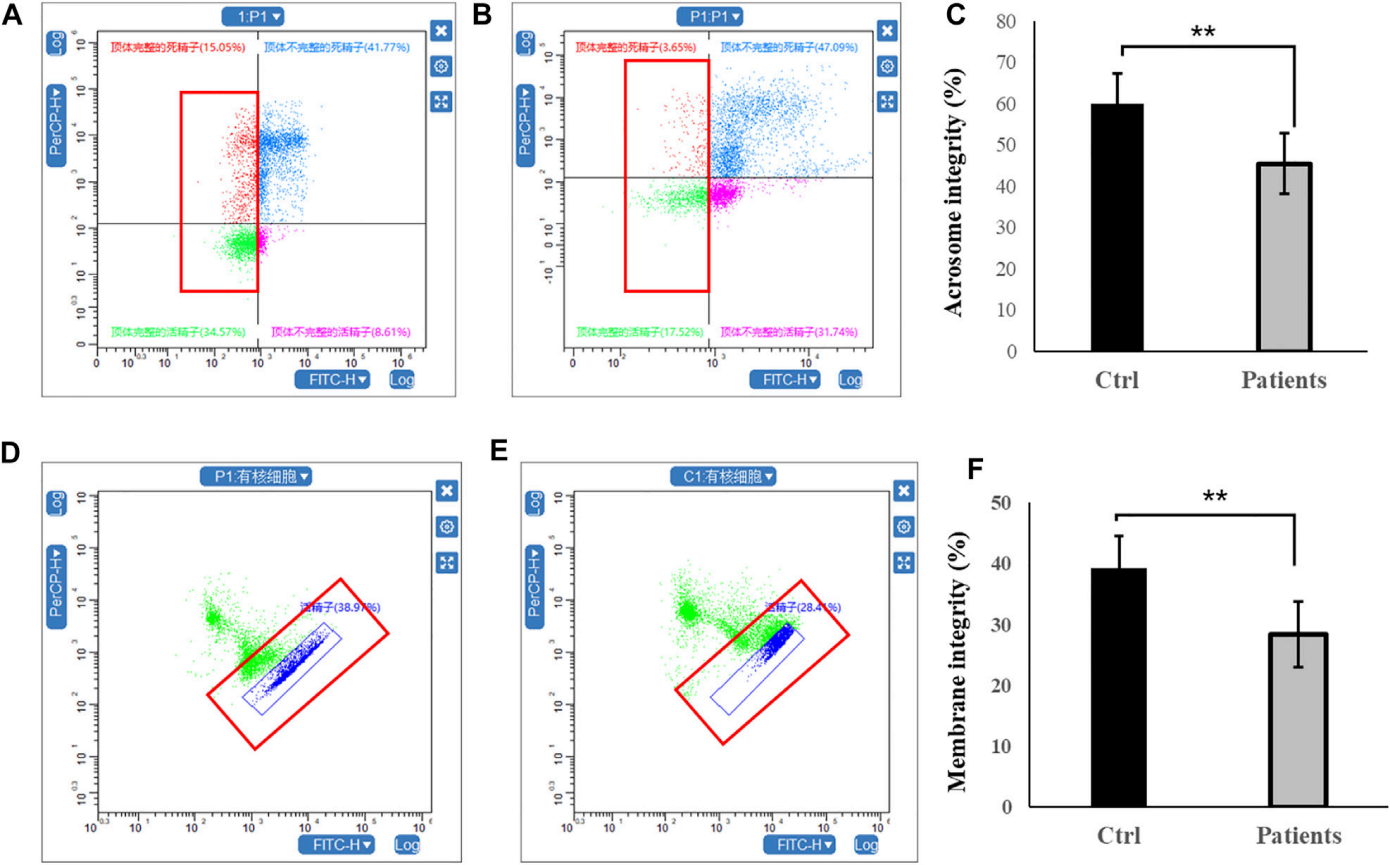
Flow cytometry analysis of acrosome integrity and membrane integrity within human spermatozoa. **(A–C)**, acrosome integrity levels. **(D–F)**, membrane integrity. All data are displayed as means ± s.e.m. (**p < 0.01, Student’s *t*-test).

Firstly, sperm acrosome integrity and membrane integrity were compared between controls and patients with bilateral varicocele by flow cytometry. There were decreases in the acrosome integrity in patients with bilateral varicocele (Figures 1A–C). Next, we analyzed membrane integrity. The results demonstrated that the membrane integrity also significantly decreased in patients with bilateral varicocele (Figures 1D–F).

### 3.3 MMP and apoptosis rate

**FIGURE 2:**
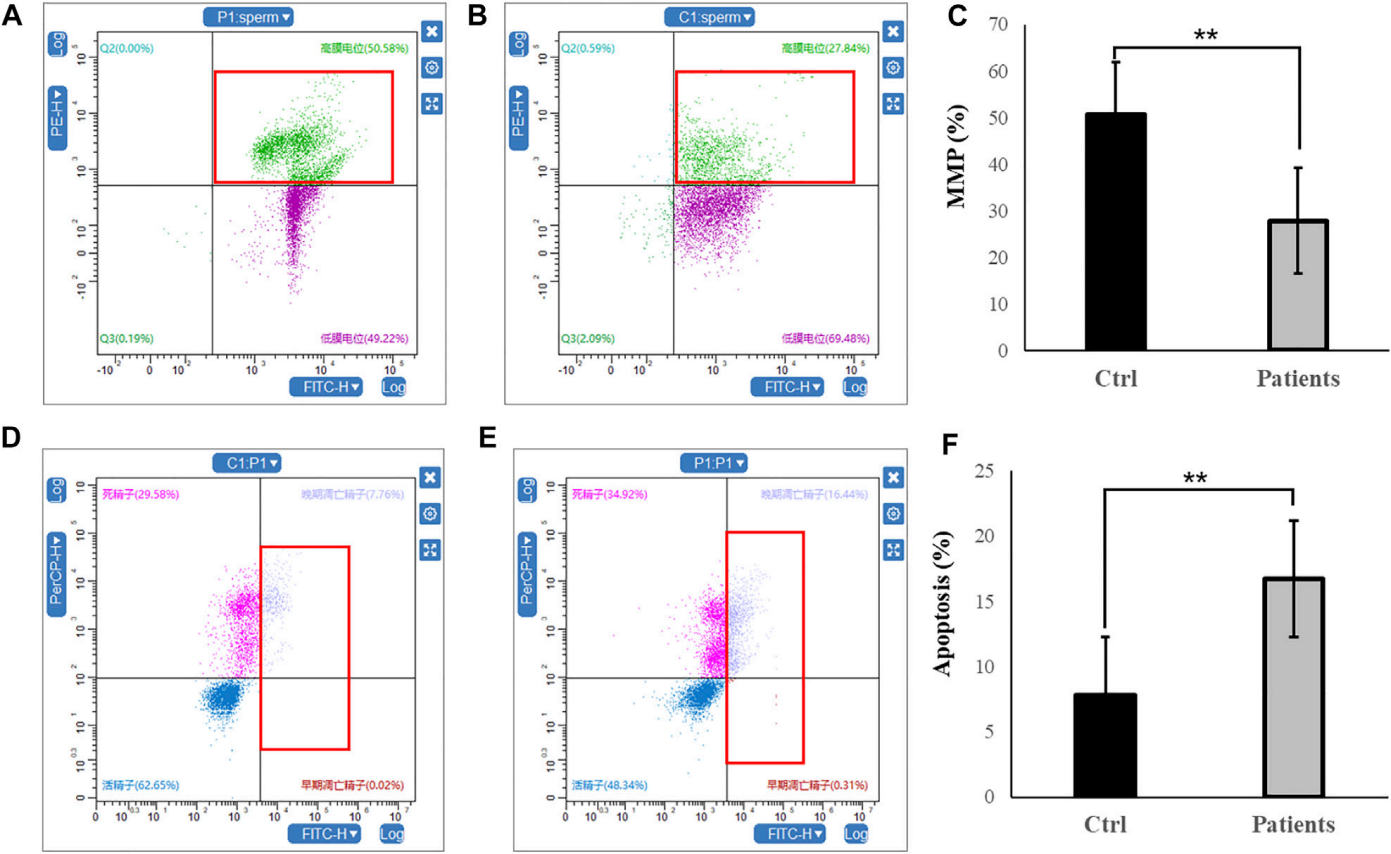
Flow cytometry analysis of MMP and apoptosis rate within human spermatozoa. **(A–C)**, MMP levels. **(D–F)**, representative flow cytometry dot plots depicting the fluorescent changes of apoptosis rate in human spermatozoa (right). All data are displayed as means ± s.e.m. (**p < 0.01, Student’s *t*-test).

In the next set of experiments, we investigated whether mitochondrial function and sperm motility are involved in MMP and apoptosis rate. Our results showed that there was ∼2-fold decrease in MMP level in patients with bilateral varicocele (Figures 2A–C). Moreover, apoptosis rate of human spermatozoa significantly (∼2.5-fold) increased in patients with bilateral varicocele (Figures 2D–F). Altogether, the results of these experiments demonstrate that MMP and apoptosis rate are involved in decreased sperm motility observed in these patients.

### 3.4 Detection of ROS and iron level

**FIGURE 3:**
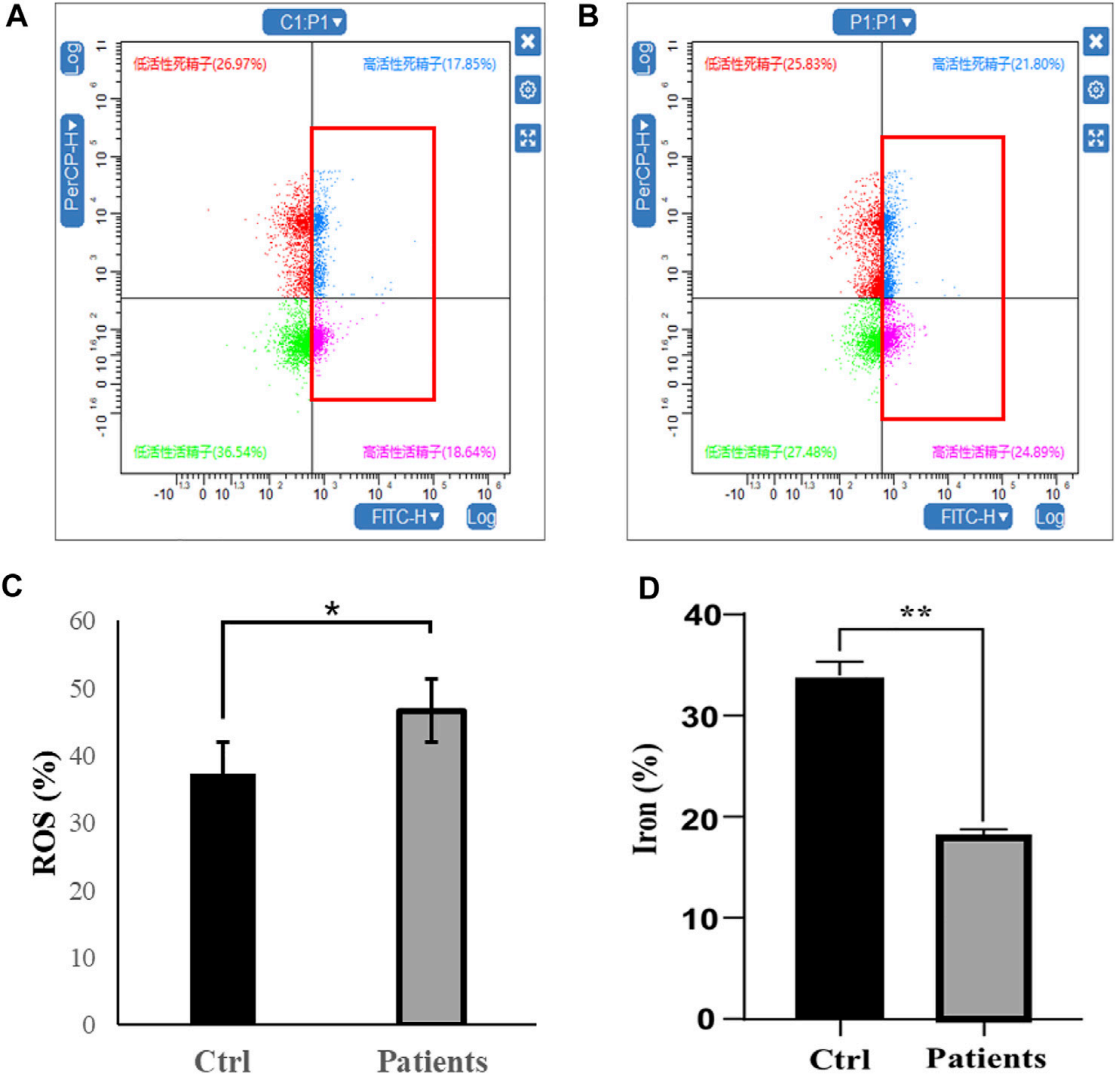
Analysis of reactive oxygen species (ROS) and iron level in human spermatozoa. **(A–C)** ROS, **(D)** iron level. All data are displayed as means ± s.e.m. (*p < 0.05, **p < 0.01; Student’s *t*-test).

To investigate the ferroptosis level between control and patients with bilateral varicocele, ROS and iron level were detected in the seminal plasma. Comparing with control group, ROS level was increased in patients with bilateral varicocele (*p* < 0.05) (Figures 3A–C). However, the level of iron was reduced in patients with bilateral varicocele (*p* < 0.01) (Figure 3D). These results together suggest that men with varicocele increased ROS levels and decreased iron levels, which might suggest the activation of the ferroptosis pathway.

### 3.5 Measurement of antioxidant enzyme activities

**FIGURE 4:**
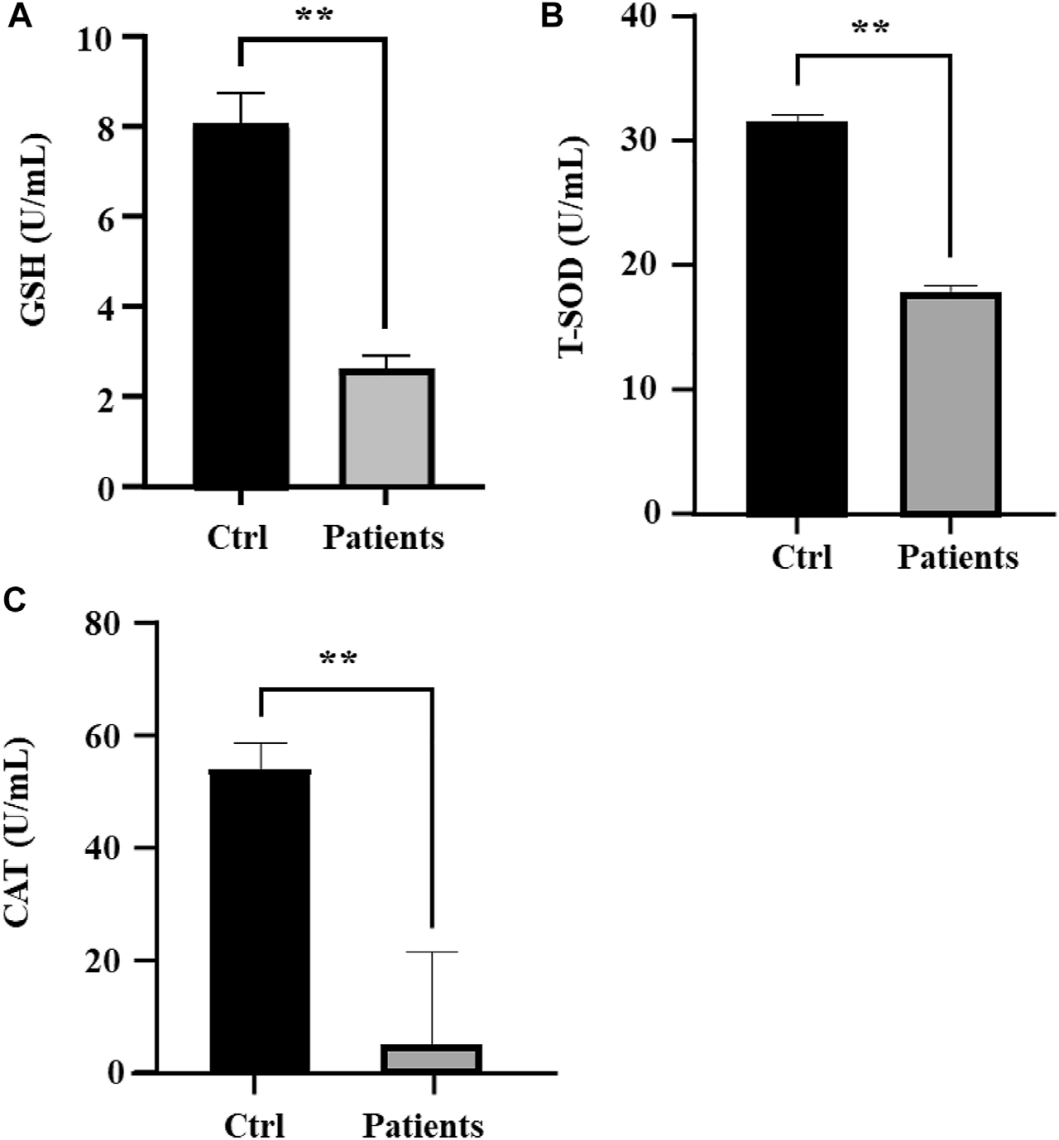
Analysis of oxidative stress in human spermatozoa. **(A)** Glutathione (GSH), **(B)** Total superoxide dismutase (T-SOD), **(C)** Catalase (CAT). All data are displayed as means ± s.e.m. (*p < 0.05, **p < 0.01; Student’s *t*-test).

Next, we investigated whether ROS and iron level in human spermatozoa affects antioxidant enzyme activities. Quantification of GSH in sperm lysates showed that there was a ∼78% (*p* < 0.01) decrease of cellular GSH in patients with bilateral varicocele (Figure 4A). T-SOD levels in patients with bilateral varicocele were decreased by ∼33% (*p* < 0.01) (Figure 4B); CAT levels in patients with bilateral varicocele were decreased by ∼95% (*p* < 0.01) (Figure 4C). According to these results, antioxidant enzyme activities were also contributed by ferroptosis.

### 3.6 ROS exposure might be associated with ferroptosis-related signal pathways

**FIGURE 5:**
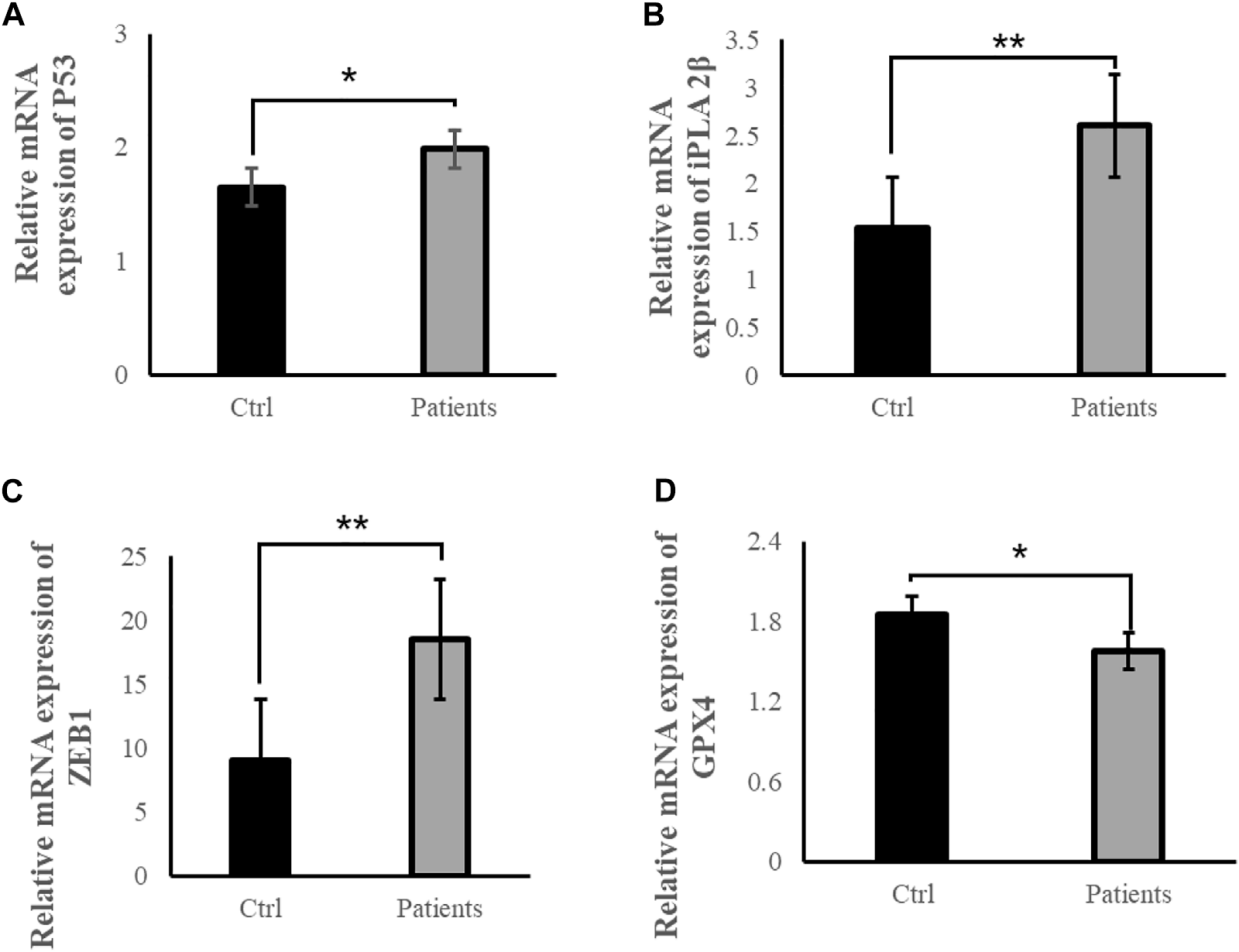
ROS exposure activated ferroptosis-related signal pathways in human spermatozoa. **(A)** Relative mRNA expression of *P53*, **(B)** Relative mRNA expression of *iPLA 2β*, **(C)** Relative mRNA expression of *ZEB1*, **(D)** Relative mRNA expression of *GPX4*. All data are displayed as means ± s.e.m. (*p < 0.05, **p < 0.01; Student’s *t*-test).

**FIGURE 6:**
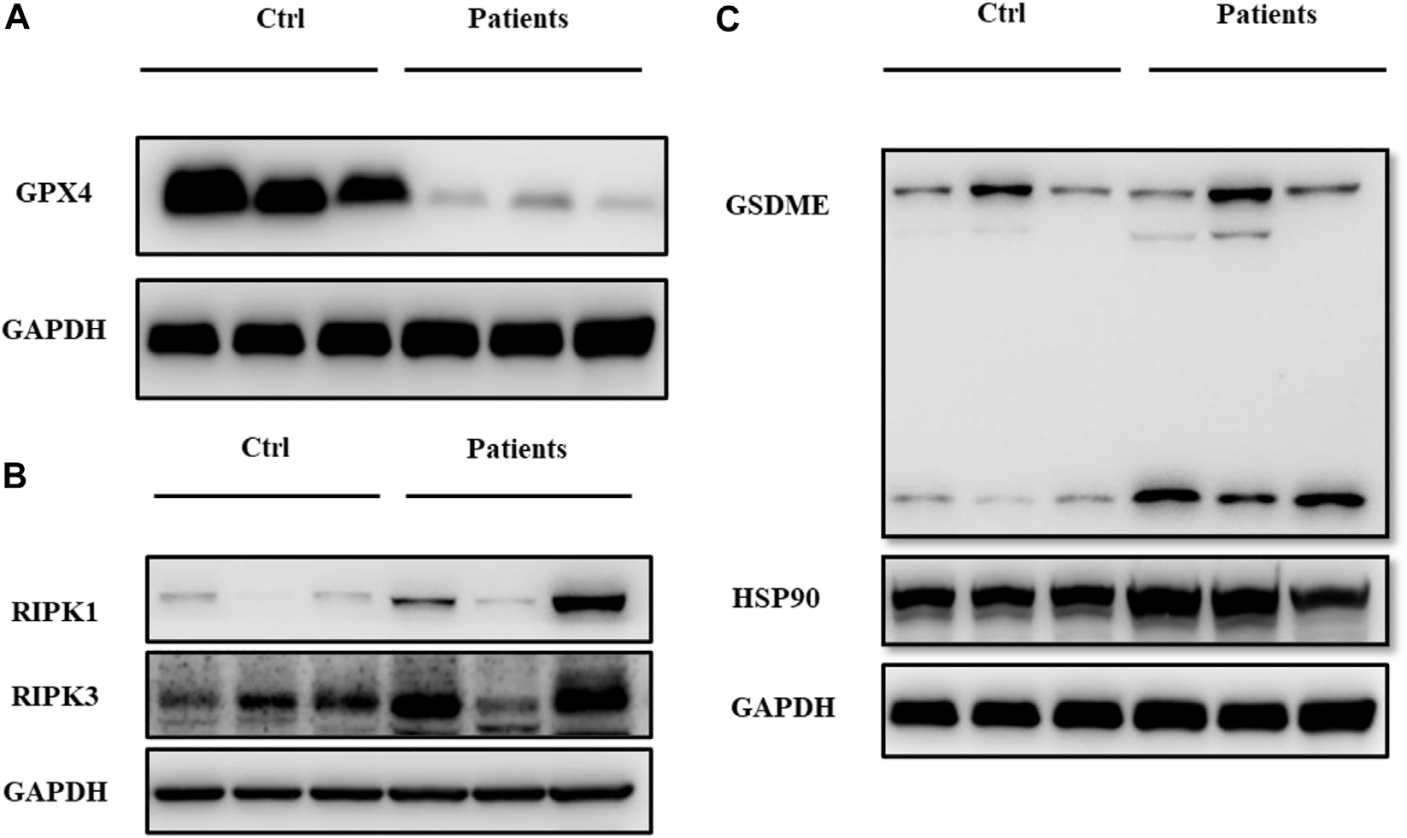
ROS exposure activated ferroptosis, necrosis and pyroptosis-related signal pathways in human spermatozoa. **(A)** Relative protein expressions of GPX4. **(B)** Relative protein expressions of RIPK1 and RIPK3. **(C)** Relative protein expressions of GSDME and HSP90.

To examine whether ROS are directly involved in ferroptosis in response to bilateral varicocele, mRNA and protein of ferroptosis-related signal pathways were detected. Quantitative real-time PCR was used to detect the mRNA expression levels of *P53*, *iPLA 2β*, *ZEB1* and *GPX4* in the semen. These results showed that the mRNA expression levels of *P53*, *iPLA 2β*, and *ZEB 1* in the patient group was significantly higher than that of the control group (*p* < 0.05) (Figures 5A–C). However, the mRNA expression level of *GPX4* in the patient group was significantly lower than that of the control group (*p* < 0.05) (Figure 5D). Besides, the level of GPX4 protein was reduced in the patient group (Figure 6A). Therefore, the results of these data demonstrate that ROS exposure might be associated with activation of ferroptosis pathway in bilateral varicocele men.

### 3.7 ROS exposure activated ferroptosis, pyroptosis and necroptosis

Except ferroptosis, we also examined whether ROS are directly involved in pyroptosis in response to bilateral varicocele. Pyroptosis and necroptosis-related proteins of RIPK1, RIPK3, and pyroptosis markers of GSDME and HSP 90 were detected. There were obvious differences in the protein expression of RIPK1, RIPK3 GSDME, and HSP 90 between two groups (Figures 6B, C). In the patients group, expression of ferroptosis, pyroptosis and necroptosis-related proteins were increased than control group.

## 4 Discussion

Bilateral varicocele is considered as a major risk factor for male infertility. Some studies have reported that bilateral varicocele can significantly decrease basic semen parameters, whereas other studies have shown that the grade and anatomical side of varicocele can affect semen quality (Vivas-Acevedo et al., 2010; Zhou et al., 2015; Teixeira et al., 2019). In the present study, we found that bilateral varicocele not only decreased sperm quality, but also affected sperm acrosome integrity, membrane integrity, MMP, apoptosis rate, ROS and iron level. However, the molecular mechanisms underlying the effects of bilateral varicocele on semen quality need further investigation.

Ferroptosis, a new programmed iron-dependent cell death identified by Brent R. Stockwell’ et al., in 2012, is associated with lipid peroxidation of ROS (Dixon et al., 2012; Ou et al., 2020). Several studies have suggested that GSH is a central player in ferroptosis and is required for GPX4 to eliminate oxidized phospholipids (Dixon et al., 2012; Imai et al., 2017; Forcina and Dixon, 2019). In this study, we found that GSH level was reduced in patients with bilateral varicocele. However, the level of ROS and iron were increased. These findings indicate that bilateral varicocele is a major factor associated with abnormalities of human semen parameters in which the ferroptosis is induced by lipid peroxidation and iron metabolism signaling, consisted with previous studies (Yang and Stockwell, 2016; Imai et al., 2017; Forcina and Dixon, 2019). To further verify the effects of bilateral varicocele and ROS on sperm quality, antioxidant enzyme activities were evaluated in this study. We found that T-SOD and CAT were reduced. Our results indicate that GSH plays a role in antioxidant defense, and the GSH-GPX4 interaction is critical for the regulation of antioxidant enzyme activities. This is consistent with the previous conclusion that GSH is associated with high level of ferroptosis and affects antioxidant enzyme activities. Previous studies have verified that lipid peroxidation is accelerated by intracellular iron (Yang and Stockwell, 2016; Forcina and Dixon, 2019). In the future, we will conduct in-depth studies on the mechanism of ferroptosis induction and the associated redox signaling pathway.

In addition, it is shown that iPLA2β (a critical regulator) mediated lipid detoxification controls p53-driven ferroptosis independent of GPX4 signal pathway (Chen et al., 2021). Our findings also indicated that ROS exposure activated ferroptosis-related signal pathways in human spermatozoa. P53 and iPLA 2β also play an important role in the regulation of ferroptosis dependent upon GPX4. The results obtained here may have implications for understanding that P53 might be related to iPLA2β, and depend on ROS increase, which reduces antioxidant enzyme activities. One study have identified that iPLA2β was a critical regulator for p53-driven ferroptosis upon ROS induced stress (Chen et al., 2021). ZEB1 is a master transcription regulator that affects ROS and oxidative stress metabolism (Han et al., 2021). A recent study indicated that ZEB1 directly inhibited GPX4 transcription, thus contributing to ROS accumulation (Han et al., 2021). These results can be considered to a significant step in the study of ferroptosis-related proteins.

Proptosis was identified for the first time by Zychlinsky et al., in 1992 and was defined as gasdermin-mediated programmed death in 2015 (Zychlinsky et al., 1992; Shi et al., 2015). One recent study indicated that Cadmium exposure induced pyroptosis (GSDME) in testicular tissue by increasing oxidative stress (Zhou et al., 2022). Another study found that HSP90 inhibitors suppressed pyroptosis and may thus become a potential therapeutic strategy (Zhou et al., 2020). However, there is no study on the relationship of pyroptosis with semen quality. We aimed to expand on the results of a previous study in which pyroptosis-related factor (GSDME) and HSP 90 were accumulated in bilateral varicocele group. These results demonstrated in this work provide a new perspective on the pyroptosis and sperm quality.

Necroptosis is a form of regulated cell death that critically depends on RIPK3 and MLKL (mixed lineage kinase domain-like) and generally manifests with morphological features of necrosis (Galluzzi et al., 2017). RIPK1 and RIPK3 are homologous kinases that are related to activation of necroptotic death (Najjar et al., 2016). The relationship of testes toxicity and RIPK1-RIPK3-MLKL signaling has been demonstrated in a recent study (Yuan et al., 2020). However, the underlying mechanism between the RIPK1-RIPK3 and semen quality has not been thoroughly studied. Some previous studies have indicated that RIPK3 is activated by RIPK1, which results in the assembly of a RIPK1- and RIPK3-containing amyloid-like signaling complex under the assistance of HSP 90 (Cho et al., 2009; He et al., 2009; Zhang et al., 2009). HSP 90 (a ubiquitous heat shock protein) is suggested as a common regulatory factor in both necroptosis and ferroptosis (Wu et al., 2019). HSP 90 may not bind directly with GPX4, but it is important for mediating the activation of RIPK1 in necroptosis (Wu et al., 2019). In our study, we found that RIPK1, RIPK3 and HSP 90 were activated in bilateral varicocele group. Therefore, these results suggest that our approach is a promising alternative to necroptosis and bilateral varicocele.

## 5 Conclusion

**FIGURE 7:**
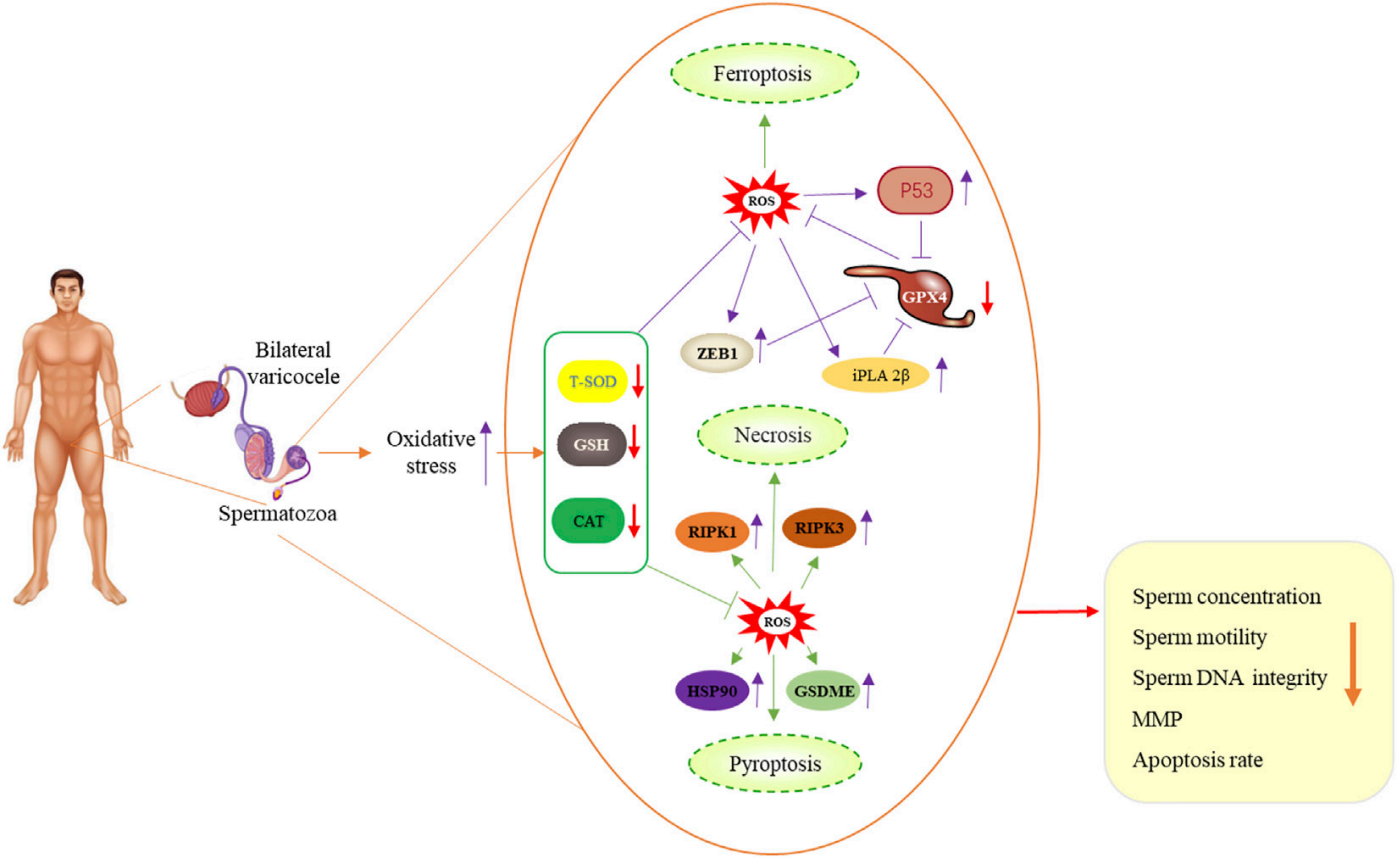
Schematic illustration of bilateral varicocele promoting ROS as well as ferroptosis, pyroptosis and necrosis in human spermatozoa.

Our results suggest that bilateral varicocele promotes ROS production and apoptosis, and induces decline of acrosome integrity, membrane integrity, MMP and antioxidative enzyme activities, indicating that it has promotive effects on ferroptosis, pyroptosis and necroptosis in human spermatozoa and affects sperm quality in infertile men (Figure 7). Based on our data, we speculate that bilateral varicocele may lead to ferroptosis, pyroptosis, necroptosis-induced cell death, and male infertility. These findings are of great importance for understanding bilateral varicocele and helping infertile/subfertile men.

## Data Availability

The original contributions presented in the study are included in the article/supplementary material, further inquiries can be directed to the corresponding authors.

## Abbreviations

CAT, Catalase; GSH, glutathione; GPX4, GSH-dependent peroxidase 4; GSDME, Gasdermin E; HSP 90, heat shock protein 90; iPLA 2β, Ca^2+^-independent phospholipases A2 beta; MMP, mitochondrial membrane potential; ROS, reactive oxygen species; RIPK1, receptor interacting serine/threonine kinase 1; RIPK3, receptor interacting serine/threonine kinase 3; T-SOD, total superoxide dismutase (T-SOD); ZEB1, Zinc finger E-box binding homeobox 1.
